# Retraction Note: Forkhead box (FOX) G1 promotes hepatocellular carcinoma epithelial-Mesenchymal transition by activating Wnt signal through forming T-cell factor-4/Beta-catenin/FOXG1 complex

**DOI:** 10.1186/s13046-023-02739-5

**Published:** 2023-06-29

**Authors:** Xingrong Zheng, Jiaxin Lin, Hewei Wu, Zhishuo Mo, Yunwen Lian, Peipei Wang, Zhaoxia Hu, Zhiliang Gao, Liang Peng, Chan Xie

**Affiliations:** 1 Department of Infectious Diseases, the Third Affiliated Hospital of Sun Yat-sen University, 600# Tianhe Road, Guangzhou, 510630 Guangdong Province China; 2 Key Laboratory of Tropical Disease Control, Ministry of Education, Sun Yat-sen University, Guangzhou, 510630 Guangdong Province China; 3 Guangdong Provincial Key Laboratory of Liver Disease, Guangzhou, China

**Retraction Note:** ***J Exp Clin Cancer Res*** **38, 475 (2019)**

10.1186/s13046-019-1433-3

The Editor in Chief has retracted this article after concerns were raised regarding a number of potential image overlap issues. Specifically:

- It appears there are several potential image overlaps between Figs. 2E and 4E of this article, despite the authors providing a correction to Fig. 2 [1].
- Further potential image overlap has been alleged between Figs. 1D and 6E of [2] by some of the same authors.
- Potential image overlap between 4B and 4B of [3] by some of the same authors.

The authors were unable to provide raw images and evidence of ethical approval. Therefore, the Editor has lost confidence in the data presented here. All authors agree to this retraction.
